# Effects of radiation therapy on the morphology and composition of root dentin and cementum in primary teeth

**DOI:** 10.1590/1807-3107bor-2025.vol39.026

**Published:** 2025-02-24

**Authors:** Julio Cesar Ramos CADILHO, Paôla Caroline da Silva MIRA, Jéssica Peixoto BEM, Penélope Swerts RODRIGUES, Francisco José de Queiroz THOMÉ, Harley Francisco de OLIVEIRA, Fernando Marin TORRES, Francisco Wanderley Garcia PAULA-SILVA, Paulo NELSON-FILHO, Alexandra Mussolino de QUEIROZ

**Affiliations:** (a) Universidade de São Paulo – USP, School of Dentistry of Ribeirão Preto, Department of Clinical Pediatrics, Ribeirão Preto, SP, Brazil.; (b) Universidade Federal de São Carlos – UFSC, School of Medicine, São Carlos, SP, Brazil.; (c) Universidade de São Paulo – USP, School of Medicine of Ribeirão Preto, Brazil.

**Keywords:** Radiotherapy, Head and Neck Neoplasms, Dentin, Dental Cementum

## Abstract

The aim of this study was to evaluate, in vitro, the effects of radiation therapy on the morphology and chemical composition of root dentin and cementum of primary teeth. Roots of human primary teeth were exposed to varying doses of irradiation using a linear accelerator: Group 1 = irradiation dose of up to 30 Gy (n= 6); Group 2 = irradiation dose of up to 42 Gy (n= 6); and Group 3 = irradiation dose of up to 54 Gy (n= 6). Non-irradiated human deciduous teeth were used as controls (n= 3). Energy-dispersive X-ray spectroscopy (EDX) data were analyzed using the chi-square test (alpha = 5%). The morphological evaluation was performed using scanning electron microscopy (SEM). In the cementum, radiation increased inorganic composition and decreased oxygen levels. However, no morphological changes were observed. In the root dentin, obliterated dentinal tubules were observed in specimens irradiated with 54 Gy, with no changes in composition. Thus, radiation therapy significantly altered the morphology and composition of important tooth structures such as dentin and cementum, in primary teeth.

## Introduction

Currently, a child dies of cancer every three minutes.^1^ Even though these data reflect a harsh reality, advances in cancer treatments and the possibility of early diagnosis of the disease have led to increased rates of survival and cure in recent decades.^2, 3^

In children, 12% of cancers occur in the head and neck region.^4^ Soft tissue sarcomas, lymphomas, and thyroid carcinomas are the most frequently observed types of primary tumors, and the most commonly affected regions are the thyroid, orbit, nasopharynx, tonsils, and paranasal sinuses.^5^ Furthermore, central nervous system tumors account for 8% to 15% of pediatric neoplasms, and they are more frequent in children under the age of 15 years.^6^

Among the modalities of head and neck cancer (HNC) treatments, radiation therapy (RT), which utilizes ionizing radiation as its therapeutic agent, has shown high success rates.^7^ Individuals undergoing RT for HNC experience several undesirable side effects, including oral fungal infections, mucositis, xerostomia, trismus, loss of taste, atrophy of the muscles of the jaw region, progressive loss of the periodontal ligament, soft tissue necrosis, osteoradionecrosis, and dental caries.^8-10^ Thus, RT seems to have a considerable impact on the oral health of patients under treatment.^10^

While RT-related carious lesions have been associated with indirect effects of irradiation,^9-11^ more recent studies have demonstrated that irradiation can directly affect the enamel and dentin surfaces in primary and permanent teeth, changing their structure^12-15^and activating enzymes involved in tissue degradation.^16, 17^These factors may contribute to a greater risk of carious lesions development after RT for the treatment of HNC.^17^

Therefore, dentists should provide care for patients with a history of cancer treatment who present carious lesions related to RT.^18^ Furthermore, given the keen interest of the World Health Organization in increasing the survival rate of children affected by cancer to 60% by 2030,^18-19^ dental treatment following RT in the head and neck region is expected to become a more frequent practice in pediatric dentistry.^18^

Understanding dental changes in these patients seems to be a crucial first step towards a restorative clinical approach based on scientific evidence. While previous studies have shown that RT can cause changes in the enamel and coronal dentin of permanent and primary human teeth,^14,15,19-21^ further studies are needed to thoroughly investigate the possible changes in the root dentin and primary cementum. This investigation is justified because primary teeth have differences in the enamel and dentin microstructure compared to permanent teeth, such as the lower thickness of these structures, the lower level of calcium and phosphate, microchannels, and larger-diameter dentinal tubules.^21,22^

Thus, the objective of this study was to evaluate, in vitro, the morphology and chemical composition of root dentin and cementum of primary teeth after irradiation with a linear accelerator, simulating head and neck radiation therapy treatment in children. The null hypothesis of this study is that irradiation would not affect the composition of root dentin and cementum of primary teeth.

## Methods

### Ethical aspects and sampling

The study was approved by the Research Ethics Committee of the Ribeirão Preto School of Dentistry, University of São Paulo (Process number: 56416516.1.0000.5419).

The sample consisted of 21 freshly extracted single- and multirooted human primary teeth, whose roots measured at least 8 mm in length, without cracks or fractures. The teeth were stored in distilled water at 4ºC.

The roots of primary teeth were irradiated using the following doses: Group 1 = up to 30 Gy (n = 6); Group 2 = up to 42 Gy (n = 6); and Group 3 = up to 54 Gy (n = 6). Non-irradiated human deciduous teeth were used as controls (n = 3).

### Teeth irradiation

The teeth were exposed to a dose fraction of 2 Gy for five consecutive days until reaching 30 Gy (n = 6), 42 Gy (n = 6), and 54 Gy (n = 8). Non-irradiated teeth (n = 3) were used as controls. A total of 27 fractions were performed for six weeks to reach the dose of 54 Gy.^21,24^ X-rays were applied using a linear accelerator (RS 2000, Rad Fonte Technologies, Inc., Suwanee, USA), operating at 200 kVp and 25 mA, equipped with a standard 0.3 mm copper filter.

The X-rays generated under this condition have a spectrum with a minimum energy of 95 kV up to 200 kV maximum energy, with a half-alue layer of 0.62 mm copper. The dose gradient of these X-rays in tissue is about 10% at a depth of 0.5 cm. The teeth were aligned equidistant from the center of the beam and inside the cone to ensure not only a uniform dose rate (approximately 2.85 Gy/min) but also a total dose delivery per fraction. During RT, the teeth were stored in distilled water, and after RT, the samples were kept in artificial saliva at 37°C.^14,24^

### Energy-dispersive X-ray spectrometry (EDX) and scanning electron microscopy (SEM)

The teeth were sectioned horizontally to separate the crowns from the roots. The roots were sectioned longitudinally (in the buccolingual direction), separating the mesial and distal roots. The sections were performed using a 0.5-mm thick diamond disc (South Bay Technology, San Clement, USA) under refrigeration at a speed of 300 rpm. The roots were then polished and prepared for SEM analysis according to the following protocol: fixation in glutaraldehyde + cacodylate, cleaning in an ultrasonic bath with distilled and deionized water for 10 minutes, dehydration in a graded ethanol series (25%, 50%, 75%, 95%, and 100%), and immersion in hexamethyldisilazane (HMDS) for 10 minutes.

After dehydration, the specimens were mounted on stubs with double-sided carbon adhesive tape and then subjected to elemental microanalysis using an energy-dispersive micro-spot X-ray spectrometer (SEM-XRF; IXRF Systems Austin, USA). The specimen surfaces were analyzed longitudinally, and X-ray counts were performed by a liquid nitrogen-cooled semiconductor Si (Li) detector. The synthetic stoichiometric hydroxyapatite reagent (Sigma-Aldrich, Sintra, Portugal), with a purity of 99.99% [Ca5HO13P3] was used as a reference. Following the acquisition of energy spectra, both phosphorus and calcium were semi-quantified (ZAF method). The data were analyzed using the chi-square test at a 5% significance level.

Before the morphological analysis, all specimens were gold-sputtered with a 20-30 nm layer in a vacuum metallization system (Bal-Tecmod. SCD 050, Fürstentum, Liechtenstein). After that, the surfaces of the specimens were analyzed morphologically to observe the changes caused in the dental tissue. Only the most representative regions were recorded. The full thickness of the root dentin and cementum was analyzed qualitatively.

## Results

### Energy-dispersive micro-spot X-ray spectrometry

In the ion composition analysis using X-ray energy-dispersive spectrometry (EDX), the percentages of oxygen (O), phosphorus (P), and calcium (Ca) in the cementum and root dentin were analyzed in irradiated and non-irradiated teeth. Table 1 shows the percentages of O, P, and Ca present in the root dentin of the control group and of the groups exposed to radiation. The differences were not statistically significant. Table 2 reveals a significant decrease in the percentage of oxygen from 42 Gy onwards, and a notable but not significant increase in the weight percentage of calcium and phosphate at the same dose. Furthermore, the percentage of O, P, and Ca present in the cementum of the control group was higher than in the groups treated with radiation.

**Table 1:** Average weight percentage (wt%) of the chemical elements present in the root dentin of irradiated and non-irradiated specimens.

Chemical elements	Control-root dentin	Group 1	Group 2	Group 3	p-value
		30 Gy	42 Gy	54 Gy	
	x	x	x	x	
Oxygen (O)	92.3 ± 2.3	95.5 ± 5.4	84.8 ± 8.3	92.8 ± 2.5	0.068
Phosphorus (P)	2.4 ± 2.1	1.3 ± 2.8	5.7 ± 2.3	2.6 ± 2.4	0.1862
Calcium (Ca)	5.3 ± 4.3	3.2 ± 5.2	9.5 ± 5.4	4.6 ± 3.7	0.1765

**Table 2:** Average weight percentage (wt%) of the chemical elements present in the root cementum of irradiated and non-irradiated specimens. Different letters in each row indicate different results.

Chemical elements	Control - Cementum	Group 1	Group 2	Group 3	p-value
		30 Gy	42 Gy	54 Gy	
	x	x	x	x	
Oxygen (O)	88.8 ± 2.2^A^	92.2 ± 2.1^A^	61.8 ± 1.5^B^	57.4 ± 2.2^C^	< 0.0001
Phosphorus (P)	3.2 ± 1.3^A^	2.4 ± 1.2^A^	10.3 ± 2.9^B^	14.4 ± 2.4^B^	0.0021
Calcium (Ca)	8.0 ± 2.9^A^	5.4 ± 3.1^A^	27.9 ± 1.2^B^	28.2 ± 1.4^B^	< 0.0001

### SEM analysis: cementum of deciduous teeth

**Figure 1:**
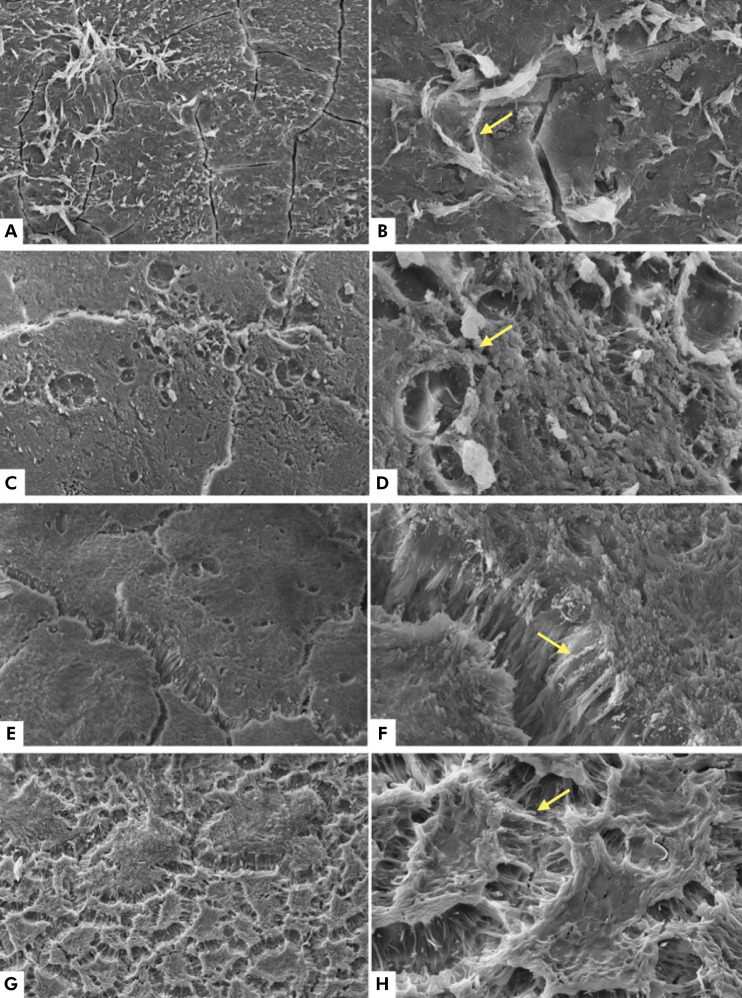
Electromicrographs of the cementum, with no visible morphological changes in the control group (A and B), at the dose of 30 Gy (C and D), at the dose of 42 Gy (E and F), and at the dose of 54 Gy (G and H). Yellow arrows indicate periodontal ligament fibers. Magnifications of 500x (A, C, E, G) and 2000x (B, D, F, H).

No changes were observed between the groups in the morphological analysis of the cementum. Fibers and organic material, indicative of the periodontal ligament, were present on the tooth surface (Figure 1).

### SEM analysis: root dentin of primary teeth

**Figure 2:**
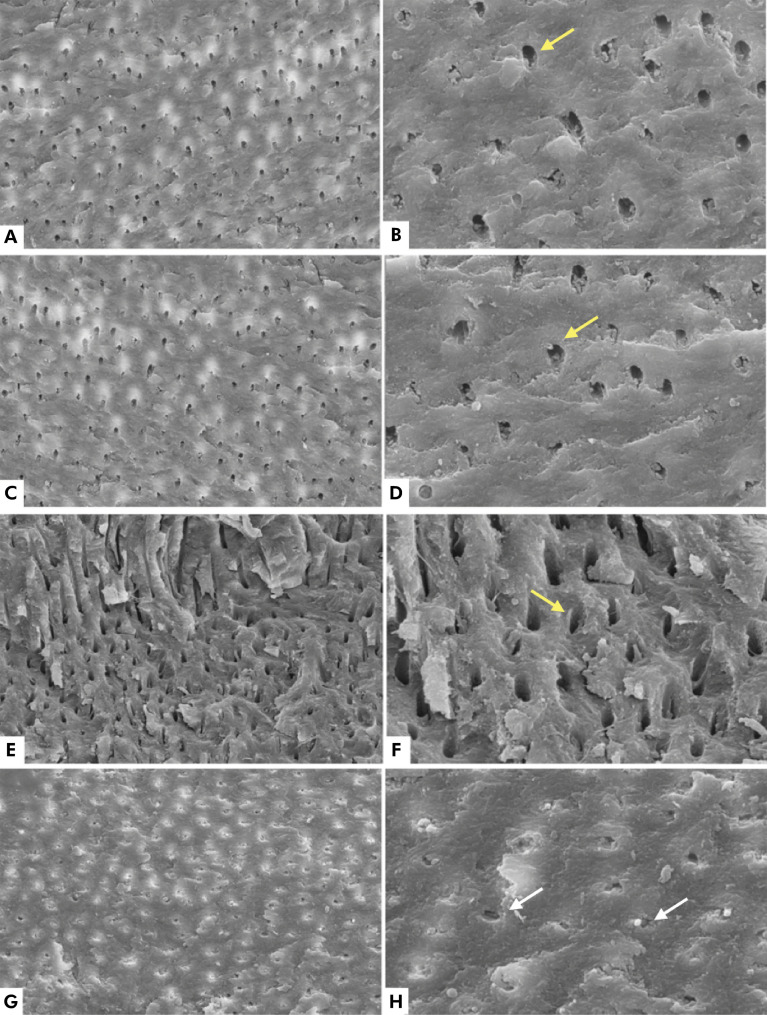
Electromicrographs of the dentin, with no visible morphological changes in the control group (A and B), at the dose of 30 Gy (C and D), and at the dose of 42 Gy (E and F). Yellow arrows indicate dentin tubules. At the dose of 54 Gy, dentin tubules became obliterated (white arrows, G and H). Magnifications of 500x (A, C, E, G) and 2000x (B, D, F, H).

In the SEM analysis of dentin morphology, the dentin of non-irradiated teeth (control) presented well-defined dentinal tubules. Also, there were no morphological changes with the increase of the irradiation dose up to 42 Gy. However, dentinal tubules were obliterated in Group 3 (irradiated up to 54 Gy) (Figure 2).

## DIscussion

The null hypothesis of this study was partially accepted, given that irradiation does not affect the composition of root dentin but changes the composition of cementum in primary teeth.

Despite the increase in pediatric cancer incidence in recent years, a higher survival rate has been noted.^2,3,18^ These trends underscore the necessity for pediatric dentists to be aware of peculiarities of patients when planning treatments.

The paucity of studies in the literature evaluating the changes caused by RT in the cementum and root dentin of primary teeth and the importance of detecting such changes motivated the development of this study. Understanding how these structures respond to RT could aid in developing strategies for endodontic treatment and in understanding possible changes during physiological root resorption (rhizolysis) in patients undergoing this type of treatment for HNC.^24-36^

RT doses for children usually range from 30 to 54 Gy. The daily dose generally is 2 Gy/day, five days a week, alternating two days without radiation, typically on the weekends, to allow for tissue recovery.^25^ Different radiation doses may contribute to treatment complications. This study evaluated the effects of different radiation doses on the tooth structure. The methodology used herein aimed to closely simulate the protocol applied to patients. Thus, between radiation cycles, the specimens were stored in artificial saliva and kept in an oven at 37°C. During irradiation, the teeth were placed in distilled water, considering that the viscosity and high concentration of ions in the artificial saliva can affect the uniform distribution of irradiation.^20,26^

Cementum is a mineralized tissue that covers the roots of human teeth and contains about 45% of inorganic substances, in addition to 55% of organic substances and water. The inorganic component consists mainly of calcium and phosphate in the form of hydroxyapatite, while the organic fraction is composed of collagen. Cementum serves to incorporate collagen fibers that connect the tooth to the alveolar bone.^27^

In this study, irradiation modified the chemical composition of the cementum. The 30 Gy dose reduced the inorganic content and increased the percentage of oxygen. At doses of 42 Gy and above, the inorganic component significantly increased, while the percentage of oxygen significantly decreased. Changes in dentin composition were not observed.

No studies have evaluated the composition of root cementum in primary teeth after irradiation. Mellara et al.^22^ assessed the chemical composition of coronal dentin after irradiation employing EDX and suggested that the decrease in oxygen may occur due to protein degradation caused by irradiation, given that the organic fraction is mainly composed of collagen.

The morphological analysis did not show changes in the cementum surface between non-irradiated and irradiated specimens, regardless of the dose. Nevertheless, changes in microhardness do not necessarily reflect morphological changes, but a reduction in cemental microhardness has been observed in permanent teeth after irradiation cycles with cobalt 60.^28^ These studies used not only different types of teeth but also different maximum radiation doses and equipment compared to those used in the present study. A direct comparison of these findings with those of other studies is not possible because of the lack of studies on morphological changes in the cementum of primary teeth caused by radiation.

Dentin consists of 70% inorganic material, 18% of organic matrix, and 12% of water (weight %), accounting for most of the tooth. The organic component is made up of collagen fibers arranged irregularly, while the inorganic component is formed by hydroxyapatite crystals.^29^In the present study, the mineral content of the root dentin was quantified with the aid of EDX, and the results obtained showed that the percentage of oxygen, phosphorus, and calcium in non-irradiated and irradiated specimens did not reveal significant changes. These findings differ from those previously obtained for coronal dentin,^15,20,23^ which showed a reduction in organic content.

Morphological changes were observed in the root dentin of primary teeth after irradiation with 54 Gy in the SEM analysis. The images indicate obliteration of the dentinal tubules. These findings are in line with those of previous studies in which obliteration of the dentinal tubules in the coronal dentin was also reported.^20,23,30^ The observed changes might have resulted from the degeneration of odontoblastic processes, disruption of intertubular dentin, and breakdown of the collagen network as direct radiation damage to cells and fibers. Irradiation leads to changes in the secondary and tertiary protein structures and has a detrimental effect on the hydration of collagen fibers.^31^

Despite methodological differences, the different types of radiation devices, and the distinct tooth substrates evaluated, most studies have observed direct radiation damage to the tooth substrate, affecting the properties of cementitious and adhesive materials relative to the dentin structure.^20,23 32-34^ Notwithstanding the limitations of the present study, we highlight the radiation-induced changes in the root dentin of primary teeth and suggest that further research could enhance the understanding of these changes in primary teeth and scientifically guide the endodontic treatment of these patients, when necessary.

## Conclusion

Radiation induced a pronounced and significant increase in the percentage of Ca and P and a lower percentage of O in the cementum. However, no marked morphological changes were noted between non-irradiated and irradiated specimens at different doses.

Some morphological changes were observed in the root dentin in the specimens irradiated with a dose of 54 Gy. The specimens exhibited well-obliterated dentin tubules without significant changes in composition.
